# Oxidized Dextran as a Macromolecular Crosslinker Stabilizes the Zein/Caseinate Nanocomplex for the Potential Oral Delivery of Curcumin

**DOI:** 10.3390/molecules24224061

**Published:** 2019-11-09

**Authors:** Nikolas J. Rodriguez, Qiaobin Hu, Yangchao Luo

**Affiliations:** Department of Nutritional Sciences, University of Connecticut, Storrs, CT 06269, USA

**Keywords:** complex nanoparticles, zein, caseinate, oxidized dextran, curcumin, encapsulation

## Abstract

In this study, we prepared complex nanoparticles from a combination of two proteins and one polysaccharide for the encapsulation and delivery of lipophilic bioactive compounds. Two proteins, zein and sodium caseinate (NaCas), provided a hydrophobic core for the encapsulation of a lipophilic compound (curcumin), while a polysaccharide dialdehyde, oxidized dextran, served as the coating material and macromolecular crosslinker to create covalent linkage with two proteins for stabilization purposes. The heating time and crosslinker concentration were optimized to achieve the desirable colloidal stability in simulated gastric and intestinal fluids. Our results suggested that heating time played a more important role than the concentration of oxidized dextran. The optimized complex nanoparticles had a particle size of around 150 nm with a PDI < 0.1 and negative surface charge. Morphological observation by transmission electron microscopy revealed a spherical shape and uniform size distribution. Fourier transform infrared and fluorescence spectroscopies evidenced the formation of Schiff base complex, confirming the validity of covalent crosslinking. Furthermore, the complex nanoparticles demonstrated superior encapsulation properties for curcumin, showing an efficiency of >90% at 10% loading. A rather slow kinetic release profile of curcumin from complex nanoparticles was observed under simulated gastrointestinal conditions. The complex nanoparticles prepared from zein, NaCas, and oxidized dextran hold promising potential for the oral delivery of lipophilic bioactive compounds.

## 1. Introduction

Biopolymer-based colloidal nanoparticles are becoming a major delivery system for various bioactive compounds due to their low toxicity, high compatibility, and easy preparation. In particular, colloidal complex nanoparticles prepared from a combination of proteins and polysaccharides have demonstrated superior physicochemical properties for encapsulation and delivery applications [1,2,3]. Nevertheless, most protein/polysaccharide complex nanoparticles are susceptible to enzymatic digestion and degradation, leading to a premature release of cargos before reaching the small intestinal for absorption [4,5].

Zein, a prolamin of corn, contains abundant hydrophobic amino acids, such as leucine, proline, and alanine [6]. As a result, zein is insoluble in water and is best dissolved in a 50–95% ethanol-aqueous solution, with 60–70% being commonly utilized. While zein is typically not found in common food products as it lacks two essential amino acids, tryptophan and lysine, it has received increasing attention for applications in the field of material sciences, including film preparation [7] and nutrient delivery systems [8], due to its unique physicochemical properties. Zein is well-known for its self-assembly behavior by a simple liquid‒liquid dispersion preparation [9], and the resultant zein nanoparticles are ideal carriers of lipophilic bioactives due to strong hydrophobic interactions [10]. To improve the colloidal stability and redispersibility of zein nanoparticles, sodium caseinate (NaCas), an amphiphilic protein from milk, is often added as a stabilizer to form complex nanoparticles with zein [11,12,13]. Since both zein and NaCas are protein-based biomacromolecules, their complex nanoparticles are highly susceptible to enzymatic digestion by proteases in the gastrointestinal (GI) tract, limiting their applications as oral delivery carriers.

Coating protein-based complex nanoparticles with polysaccharide is commonly studied to improve the GI-stability for oral delivery applications. During the past few years, our lab has developed a series of polysaccharide-coated protein complex nanoparticles [10,14,15]. We found that the type of polysaccharide and the use of chemical crosslinkers are the two major factors determining the GI-stability improvement. For instance, our previous study revealed that coating zein/NaCas nanoparticles with pectin and crosslinking the structure by creating covalent linkage significantly improved the colloidal stability as well as the controlled release profile under simulated GI conditions [16]. Recently, oxidized dextran, a polysaccharide dialdehyde, has been exploited to function simultaneously as a coating material and a macromolecular crosslinker for stabilizing protein-based and lipid-based complex nanoparticles [10,17]. Dextran dialdehyde is prepared from the oxidation of dextran by sodium periodate and had previously been used for food and pharmaceutical applications without toxicity concerns [18,19], overcoming the limitations from synthetic and small molecular crosslinkers, such as glutaraldehyde.

Since the chemical reaction between oxidized dextran and proteins is the formation of Schiff base complex by creating covalent linkage via imine bonds, it becomes essential to optimize the reaction when different proteins are used to prepare complex nanoparticles [20]. The major objective of this study was to optimize the preparation parameters, including heating time and oxidized dextran concentration, to fabricate crosslinked zein/NaCas/oxidized dextran complex nanoparticles for encapsulation and delivery of curcumin, studied as a model lipophilic bioactive compound. An array of characterization tools was utilized to study the physical and chemical properties of as-prepared complex nanoparticles.

## 2. Results and Discussion

### 2.1. Particulate Properties and Colloidal Stability

**Figure 1:**
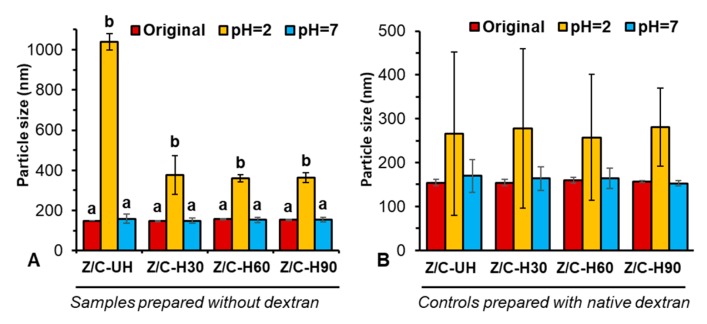
Characterization of biopolymer complex nanoparticles prepared without dextran (**A**) and with native dextran (**B**). Abbreviations: Z/C-UH, zein/NaCas nanoparticles without heating; Z/C-H30, -H60 and -H90: zein/NaCas nanoparticles with heating under 80 °C for 30, 60, and 90 min, respectively. Under each heating treatment, sample with different letters were significantly different at the level of *p* < 0.05.

All freshly prepared nanoparticles without dextran (Figure 1A) exhibited a particle size of roughly 145 nm. These nanoparticles had poor colloidal stability during the stability test under simulated gastric fluid (SGF), showing significantly increased particle size, although they were generally stable under simulated intestinal fluid (SIF) condition. In particular, visible aggregates with a dimension of >1 µm were formed if the nanoparticles were prepared without heating. On the other hand, the size of heated nanoparticles increased to around 350 nm, a significant improvement in their stability under SGF compared to the unheated sample. However, the extended heating time did not further improve the stability.

Another set of control nanoparticles was also prepared with native dextran to explore the necessity of using oxidized dextran (Figure 1B). By coating nanoparticles with native dextran, the size of complex nanoparticles increased by 5 nm, which might correspond to the thickness of the dextran surface coating. Indeed, the colloidal stability of these coated complex nanoparticles under SGF was greatly improved. After incubation in SGF, all samples showed a particle size of roughly 260 nm, and interestingly, the heating process had no impact on the stability. Nevertheless, a dramatically higher standard deviation of hydrodynamic dimension among measurements was observed for all nanoparticles after incubation under SGF, with the size ranging from 75 to 450 nm and thus a PDI of 0.19–0.48. This was indicative of a heterogeneous distribution of particle size, probably due to the loose dextran coating on the surface of the zein/NaCas nanoparticles.

When coated and crosslinked with oxidized dextran (Table 1), the complex nanoparticles exhibited a particle size of roughly 150 nm with a PDI < 0.1, across all preparation conditions, i.e., two concentrations of oxidized dextran and three heating durations, indicating a homogenous distribution in hydrodynamic dimensions. Longer heating time (60 and 90 min vs. 30 min) increased count rate of dynamic light scattering (DLS) signals of complex nanoparticles (data not shown), suggesting the existence of a higher concentration of colloidal nanoparticles as induced by heating [21,22]. Zeta potential of all samples maintained relatively stable around −36 to −40 mV (data not shown). Unlike the nanoparticles prepared with native dextran, heating process played a significant role for the GI-stability of nanoparticles prepared with oxidized dextran. Under SGF conditions, regardless of the concentration of oxidized dextran, the unheated samples exhibited poor stability with a particle size between 570 and 580 nm, a dramatic increase from their respective original sizes. Conversely, nanoparticles prepared with heating demonstrated superior colloidal stability under SGF. Nanoparticles that were heated for 30 min exhibited an increase of roughly 20–30 nm, while nanoparticles that were heated for 60 and 90 min almost maintained their original particle size, with no significant changes before and after incubation. Nevertheless, the concentration of oxidized dextran seemed to have no major influence on the stability, compared to the heating process. All nanoparticles (without or with heating) had exceptional stability under SIF conditions, although the size was reduced to around 100 nm, which was possibly due to the screening effect from high ionic strength causing the shrinkage in hydrodynamic dimensions. In addition, the PDI of nanoparticles prepared with heating was generally maintained under 0.3, a cutoff value between monodistribution and polydistribution.

### 2.2. FTIR Study

**Figure 2:**
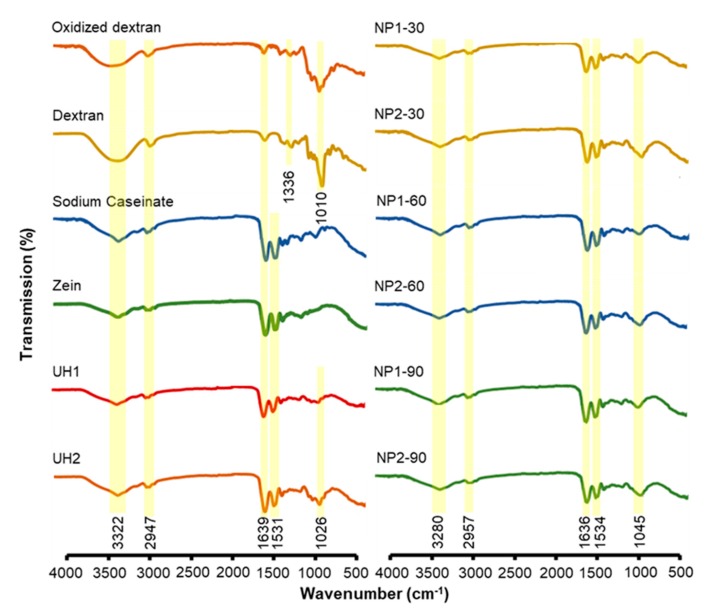
FT-IR spectrum of individual biopolymer and various nanoparticles prepared under different conditions and formulations.

Figure 2 shows the FTIR spectra for each polymer and their complex nanoparticles prepared under different conditions. Both oxidized and native dextran had a broad OH stretching around 3322 cm^−1^, showing strong hydrophilic properties, while oxidized dextran exhibited a much weaker intensity than native dextran. This may be ascribed to periodate oxidation on the hydroxyl groups. Nevertheless, oxidized dextran did not show a peak around 1730 cm^−1^, a characteristic peak of carbonyl stretching from aldehyde groups. Our result was corroborated by several other studies and may be explained by a low oxidation degree and formation of hemiacetals [23]. In addition, our previous study reported the appearance of aldehyde groups at 1730 cm^−1^, when the oxidation degree was high [10] and in another study the successful modification with dialdehyde groups with a low oxidation degree under the same preparation condition was validated by the nuclear magnetic resonance spectrum [17]. Zein and NaCas exhibited common protein characteristic peaks of hydrogen bonds, amide I and amide II groups [14,16]. The stretching of Schiff base formation [24] in the range of 1036–1639 cm^−1^ was observed in all complex nanoparticles prepared with oxidized dextran, both unheated and heated samples. However, this peak overlapped with the amide bonds present in two proteins. It is also worth noting that all the complex nanoparticles prepared with heating process exhibited a peak at 1045 cm^−1^, while samples prepared without heating showed characteristic peak at 1026 cm^−1^. Such a shift in C‒O stretching may be ascribed to the skeletal changes in polymeric structure induced by heating treatment.

### 2.3. Fluorescence Study

**Figure 3:**
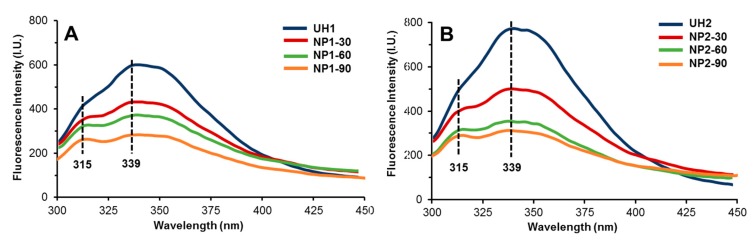
Intrinsic fluorescence property of compared nanoparticles prepared with different amount oxidized dextran, i.e., 1 mg/mL (**A**) and 2 mg/mL (**B**).

NaCas and zein are rich in tryptophan and tyrosine, respectively, the two major amino acid fluorophores providing the intrinsic fluorescence property. The changes in intrinsic fluorescence intensity are commonly used to probe the molecular interactions between the protein and other molecules in a colloidal system. In particular, fluorescence quenching, often induced by a variety of molecular interactions, can be either dynamic (resulting from collisions between the fluorophore and quencher) or static (resulting from the formation of a complex between the fluorophore and quencher) [25]. When excited at 280 nm, a broad peak of emission band was observed at 339 nm for samples prepared with both concentrations of oxidized dextran (Figure 3). This peak is known for intrinsic fluorescence from tryptophan residues, and thus the changes in its intensity are primarily due to the alteration of the micro-environment surrounding this particular amino acid. A significant reduction in the fluorescence intensity at 339 nm was observed in heated samples, which we speculated to be associated with the polarity of tryptophan micro-environment. This change may be induced by two types of interactions, i.e., (1) the heating-induced hydrophobic interactions that triggers conformational changes in protein structure and (2) the formation of a Schiff base complex between the oxidized dextran and two proteins. Consistent with our previous study [14], stronger fluorescence intensity was noticed in the unheated sample prepared with a high concentration of oxidized dextran (UH2). As a hydrophilic polysaccharide, oxidized dextran may suppress the collision between zein and NaCas during the liquid‒liquid dispersion and thus attenuate the dynamic quenching effect. As a result, a higher concentration of oxidized dextran led to the stronger intrinsic fluorescence intensity. Interestingly, a distinct peak at 315 nm was noted for the heated samples, and the peak became sharper as the heating time increased. This might be pertinent to the formation of complex structure around tyrosine residues in zein upon extended heating [26], although further study is needed to investigate this mechanistically.

### 2.4. Morphology of Freshly Prepared Nanoparticles

**Figure 4:**
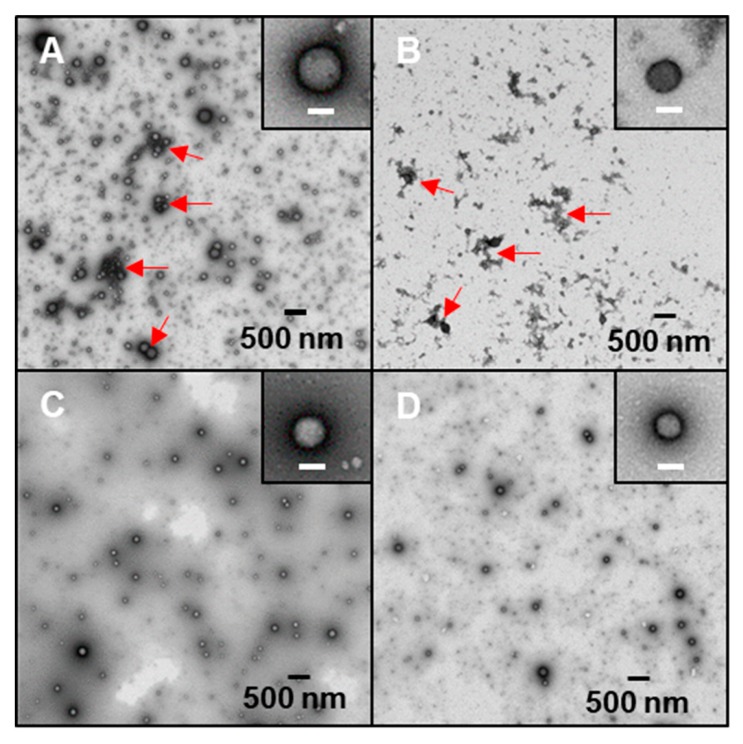
TEM images of freshly prepared nanoparticles.(**A**) UH1 (unheated complex nanoparticles with low concentration of oxidized dextran), (**B**) and (**C**) NP1-30 and NP1-60 (30 and 60 min-heated complex nanoparticles with low concentration of oxidized dextran, respectively), (**D**) NP2-60 (60 min-heated complex nanoparticles with high concentration of oxidized dextran).

Freshly prepared colloidal samples were subject to morphological observation under TEM. It is notable that visible aggregates with irregular shape were found in both the unheated (UH1) and 30 min heated (NP1-30) complex nanoparticles, as indicated by red arrows in Figure 4A,B, respectively. When samples were heated for 60 min or longer (90 min), such aggregates were not observed in complex nanoparticles prepared with either low (NP1-60, Figure 4C) or high (NP2-60, Figure 4D) concentration of oxidized dextran. NP2 samples (data of UH2 and NP2-30 were not shown), which were prepared with high concentration of oxidized dextran, showed nearly identical morphology of irregular shaped aggregates, as observed in their counterparts of UH1 and NP1-30 samples. This observation revealed that heating time played a more important role in determining the colloidal morphology than the concentration of oxidized dextran during the preparation of complex nanoparticles. It is conceivable that with a higher concentration of oxidized dextran, a longer heating time would be needed for the complete conjugation reaction. However, our results suggested that a heating time of 60 min was sufficient to stabilize the complex nanoparticles prepared with both concentrations of oxidized dextran.

Based on the DLS results discussed above, along with TEM observation, NP1-60 and NP2-60 samples were chosen for further study to explore whether a higher concentration of oxidized dextran may have an impact on the drying and redispersibility of complex nanoparticles.

### 2.5. Spray-Drying and Redispersion of Nanoparticles

**Figure 5:**
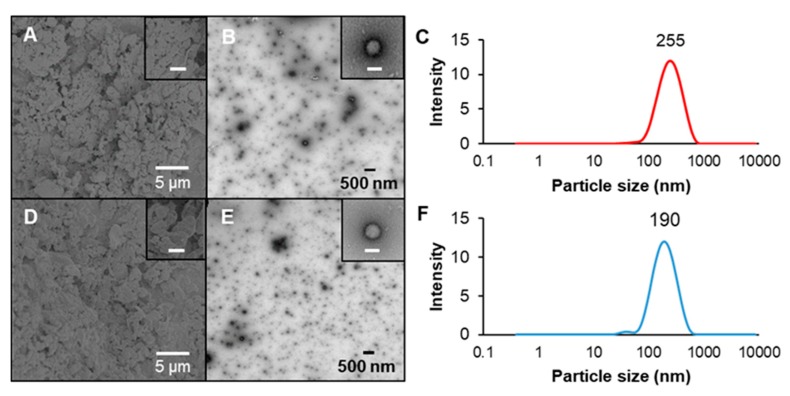
Characterization of spray-dried and redispersed nanoparticles. (**A**) and (**D**), SEM images of spray-dried powder; (**B**) and (**E**), TEM images of redispersed nanoparticles, along with (**C**) and (**F**), their respective size distribution measured by DLS. The (**A**–**C**) and (**D**–**F**) are obtained from nanoparticles prepared low and high concentration of oxidized dextran with 60 min heating time, i.e., NP1-60 and NP2-60, respectively. The scale bars in the inserted images in (**A**) and (**D**) are 2.5 µm, and in (**B**) and (**E**) they are 250 nm.

NP1-60 and NP2-60 samples were spray-dried to evaluate their size and morphology in the dried state as well as the colloidal state after the powder being redispersed into water. Appreciable agglomerates were observed after spray-drying, without a distinguishable difference in the morphology between nanoparticles prepared with two concentrations of oxidized dextran (Figure 5A,D). Interestingly, NP1-60 sample with a low concentration of crosslinker showed a larger particle size (255 nm) than NP2-60 sample (190 nm) after their powders were redispersed into water at the same concentration, as evidenced by TEM images (Figure 5B,E) and DLS size distribution data (Figure 5C,F). Formation of large agglomerates or aggregates is commonly reported for spray-dried protein/polysaccharide complex nanoparticles [10,27]. Whether such agglomerates or aggregates are able to break up and regain their original nanoscale structures upon redispersion into water is contingent on their composition and interactions [28,29]. Covalent interactions created between polymeric compositions are a critical consideration. Our previous study revealed that the use of synthetic and small molecular chemical crosslinkers compromised the redispersibility of biopolymer complex nanoparticles [16]. Our current work suggested that using oxidized dextran as a macromolecular crosslinker to strengthen the polymer network, however, did not significantly alter the original nanoscale and particulate structure upon redispersion of spray-dried powder in water. NaCas has been commonly used as a stabilizer to improve the redispersibility of hydrophobic zein nanoparticles [30,31], while these protein-based complex nanoparticles are still subject to premature degradation by digestive enzymes in the stomach, making them unsuitable for oral delivery applications. The addition of oxidized dextran as a coating and crosslinker not only greatly improved the GI-stability of zein/NaCas complex nanoparticles, but also maximally retained the colloidal nanostructure after drying and redispersion.

### 2.6. Encapsulation and Kinetic Release Profile of Curcumin

Two methods, pH-driven dissolution in NaCas (Method 1) and ethanol-dissolution in zein (Method 2), were adapted to load curcumin into the complex nanoparticles. As shown in Table 2, both loading methods exhibited very high encapsulation efficiencies of >90% at 10% loading ratio. However, the nanoparticles prepared with Method 2 exhibited dramatically better characteristics, especially particle size and PDI, which were very similar to their original empty nanoparticles without curcumin encapsulation. Thus, complex nanoparticles prepared with Method 2 were chosen due to a smaller particle size and a more reliable count rate with less deviation among replicates (data not shown). Loading a lipophilic compound into the core of nanocarrier is a major consideration during the fabrication of delivery systems. Our previous study compared a variety of methods to load curcumin into NaCas-emulsified solid lipid nanoparticles [32], and we found that pH-driven dissolution in NaCas was actually a better approach than pre-dissolving curcumin in ethanol. Pre-encapsulating curcumin into the NaCas micellar structure could help it migrate into the solid lipid core during the following emulsification process. In the current study, since the hydrophobic zein protein served as the hydrophobic core of complex nanoparticles, it was conceivable that using ethanol as a co-solvent for co-dissolving curcumin and zein facilitated curcumin encapsulation during the subsequent liquid‒liquid dispersion process. Conversely, if curcumin was pre-encapsulated into the NaCas micellar structure, it was hard for curcumin to migrate into zein nanoparticles, where were formed immediately upon liquid-liquid dispersion. Thus, we speculated that the significantly larger particle size resulting from Method 1 preparation could be ascribed to the encapsulation of curcumin primarily in the NaCas coating layer, rather than in the core of zein nanoparticles. For both methods, the nanoparticles prepared with high concentration of oxidized dextran had a slightly greater encapsulation.

**Figure 6:**
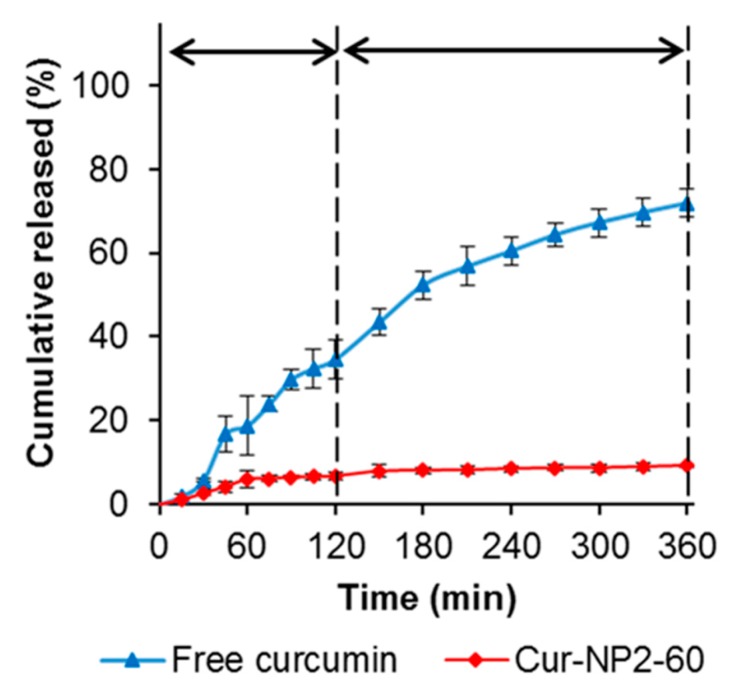
Controlled release of curcumin from nanoparticles under simulated gastrointestinal pH conditions.

The kinetic release profile of curcumin from Cur-NP2-60 was evaluated under simulated GI fluids, with free curcumin as the control (Figure 6). The diffusion rate of free curcumin was at a constant and rapid rate in both SGF and SIF conditions, reaching about 70% cumulative release at the end of incubation. However, after encapsulation into complex nanoparticles, the release rate of curcumin was significantly slower and only about 10% of total curcumin was released throughout the entire SGF and SIF incubation period. This observation was very similar to our previous study on the oxidized dextran crosslinked chitosan/NaCas complex nanoparticles [17]. This kinetic release profile was significantly slower than most of other colloidal systems, including solid lipid nanoparticles [3,33], polysaccharide/protein complex nanoparticles [34,35], and nano-in-hydrogel beads [36,37]. It will be interesting to investigate how these complex nanoparticles can be absorbed through small intestinal mucosal and carry curcumin into the circulation.

## 3. Materials and Methods

### 3.1. Materials

Dextran (40 kDa) was obtained from Alfa Aesar. Sodium (meta)periodate (NaIO_4_), sodium caseinate (NaCas), and zein were obtained from Sigma-Aldrich (St. Louis, MO, USA). Curcumin (98% purity) was obtained from ACROS Organic (Geel, Belgium). Pepsin and pancreatin were purchased from Fiscer Scientific (Pittsburgh, PA, USA). All chemicals were of analytical grade and used as received.

### 3.2. Preparation of Oxidized Dextran, Zein, and Sodium Caseinate

Oxidized dextran was prepared with modifications, as described elsewhere [38]. Briefly, 1.65 g of dextran (40 kDa) were dissolved in 50 mL of water, followed by the addition of 3.85 g of NaIO_4_ for a reaction at room temperature for 90 min under gentle stirring in the dark. The reactant was dialyzed (12–14 kDa molecular weight cutoff) against 5 L water at room temperature under constant stirring for 10 h. The dialysis medium was changed every 2 h for a total of five times. The solution was then frozen for 24 h and lyophilized using FreeZone Console Freeze Dry System (Labconco, Kansas City, MO, USA). The obtained powder was dissolved in water as a stock solution of 5 mg/mL for use in the preparation of complex nanoparticles.

Zein (10 mg/mL) was prepared in 70% aqueous ethanol solvent, while NaCas was prepared in pure H_2_O at 10 mg/mL. Both solutions were centrifuged at 8000 g for 15 min to remove undissolved impurities. Zein and NaCas solutions were prepared freshly every day prior to use.

### 3.3. Preparation of Complex Nanoparticles

To prepare zein/NaCas complex nanoparticles, 7 mL H_2_O, 2 mL NaCas and 1 mL zein stock solutions were added into a glass vial in sequential order while stirring. The total biopolymer concentration in these samples was 3 mg/mL. The samples were allowed to stir for 10 min before adjusting the pH to 6.2. Subsequently, four heating treatments were tested: unheated (Z/C-UH), and 30 (Z/C-30), 60 (Z/C-60) and 90 (Z/C-30) min heating at 80 °C while being stirred.

For samples with oxidized dextran as a macromolecular crosslinker, the same protocols mentioned above were followed to prepare zein/NaCas nanoparticles, but we replaced 1 or 2 mL H_2_O with oxidized dextran (5 mg/mL). The final samples were designated as NP1-30, NP1-60, NP1-90 (prepared with 1 mL of oxidized dextran, low concentration), and NP2-30, NP2-60, NP2-90 (prepared with 2 mL of oxidized dextran, high concentration). In parallel, in each respective heating treatment, control nanoparticles were also prepared with zein/NaCas complex and native dextran, which were labeled as Z/C/D-UH, Z/C/D-30, Z/C/D-60, and Z/C/D-90.

### 3.4. Particulate Properties

Using dynamic light scattering (DLS) via a Malvern Nano ZetaSizer ZS model (Malvern Instruments, Ltd., Malvern, UK), freshly made nanoparticles were measured for their particle size, count rate, and polydispersity index (PDI). Samples were diluted five times with water prior to measurement to meet the sensitivity requirements of the instrument. The zeta potential was also determined by the same instrument via laser Doppler microelectrophoresis technology.

### 3.5. Intrinsic Fluorescence Property

The fluorescence spectra of different samples were captured at an excitation wavelength of 280 nm with scanning from 300 to 450 nm for the emission wavelength range, using a PerkinElmer LS55 fluorescence spectrophotometer (PerkinElmer, Waltham, MA, USA). Samples were diluted appropriately to suit the sensitivity of the instrument.

### 3.6. Fourier Transform Infrared Spectroscopy

Fourier transform infrared Spectroscopy (FTIR) analysis was performed by a Nicolet iS5 FTIR spectrophotometer (Thermo Fisher Scientific, Waltham, MA, USA). OMNIC software version 8.0 was used to analyze the spectra data. All ingredients were air-dried on a disposable aluminum pan, which was then measured directly using an attenuated total reflection (ATR) accessory. The spectra were obtained by scanning from 500 to 4000 cm^−1^ with a resolution of 4 cm^−1^.

### 3.7. Stability in Simulated Gastric and Intestinal Fluid

Freshly prepared complex nanoparticles were tested for their colloidal stability in simulated gastric fluid (SGF) and simulated intestinal fluid (SIF). Briefly, 0.1 mL of each sample was mixed with 0.9 mL of either SGF mixed with pepsin (1 mg/mL) or SIF mixed with pancreatin (10 mg/mL) and incubated at 37 °C for either 2 or 4 h, respectively. Using a Malvern Nano ZetaSizer ZS model (Malvern Instruments, Ltd.), particle size and PDI were measured after the incubation to check the colloidal stability.

### 3.8. Morphological Observation

Morphology of freshly prepared original samples and redispersed samples was observed under a transmission electron microscope (TEM). Firstly, diluted samples were loaded onto a plasma-cleaned carbon TEM grid (CF400-CU, Electron Microscopy Science, Hatfield, PA, USA) and stained with 0.5% uranyl acetate stain solution. Then, the morphology was captured by a TEM (FEI, Tecnai 12 G2, Spirit, BioTWIN, Eindhoven, Netherlands) equipped with a CCD camera (AMT 2k XR40). For the spray-dried powder samples, the morphology was observed by a scanning electron microscope (SEM, JSM-6330F, JEOL Ltd., Tokyo, Japan), by mounting the powder on a double-adhesive carbon tape pre-affixed on a specimen stub.

### 3.9. Spray-Drying and Redispersion

For spray-drying, a Nano Spray Dryer B90 (Büchi Labortechnik AG, Flawil, Switzerland) was used with the following operation parameters: feeding flow rate of 120 L/min, an inlet temperature of 100 °C, and a mesh size of 5.5 μm. Freshly prepared nanoparticles were spray-dried into a fine powder, then redispersed at a 10 times dilution of the original samples to check the redispersibility by determining the particulate properties as described in Section 3.4.

### 3.10. Encapsulation and Release Studies

Two methods were adopted to encapsulate curcumin into the complex nanoparticles and the particulate properties of the resulting curcumin-loaded nanoparticles were compared. In Method 1, curcumin was first mixed with NaCas, while in Method 2, curcumin was premixed with the zein solution. Formulations of NP1-60 and NP2-60 were selected to study the encapsulation and release properties, based on previous characterizations. Method 1 was adopted from Pan et al. [39] with minor modifications. In this method, curcumin powder was directly added to NaCas at 10% loading by weight of either NP1-60 or NP2-60. Then, the pH of NaCas and curcumin mixture was adjusted to pH 12 to solubilize curcumin by protonation. The mixture was gently stirred for 30 min to allow complete solubilization prior to adjusting the pH back to 7. Samples were then centrifuged at 6000 *g* for 10 min to remove any precipitates (free and undissolved curcumin). The supernatant NaCas/curcumin mixture was used to prepare complex nanoparticles following the same preparation protocols described in Section 3.3. In Method 2, instead, curcumin powder at 10% by weight of either NP1-60 or NP2-60 was added to the zein solution in 70% aqueous ethanol. The zein/curcumin solution was stirred for 30 min prior to being used for the preparation of complex nanoparticles.

The encapsulation efficiency of curcumin in complex nanoparticles was determined using the method described in our previous study [17]. Samples were ultra-filtered using Amicon^®^ Ultra centrifugal filters (Merck KGaA, Darmstadt, Germany) with a 10,000 molecular weight cutoff at 6000 *g* for 20 min. The concentration of ultra-filtered curcumin in the receiver compartment was determined by measuring absorbance at 430 nm using a UV-Vis spectrophotometer (Evolution 201, Thermo Fisher Scientific). The encapsulation efficiency (EE) was calculated using the following Equation (1):

(1) $$\mathrm{EE}\;\left(\%\right)\;=\;\frac{\mathrm{Total}\;\mathrm{curcumin}-\text{ultra-filtrated}\;\mathrm{curcumin}}{\mathrm{Total}\;\mathrm{curcumin}}\times 100\%.$$

The controlled release profile was evaluated under SGF and SIF, consecutively. Briefly, 3 mL of freshly prepared curcumin-loaded nanoparticles were placed into a dialysis bag (12–14 kDa molecular weight cutoff) and submerged in 60 mL of SGF/ethanol solution (50:50 *v*/*v*) at 37 °C for 2 h. Ethanol was added to create a sink condition for curcumin to permeate the dialysis membrane [32,40,41]. After 2 h, samples were submerged in 60 mL of SIF/ethanol solution (50:50 *v*/*v*) at the same temperature for 4 h. At predetermined time intervals, 1 mL of release medium was withdrawn and the curcumin concentration was determined spectrophotometrically as described above. An equivalent volume of fresh release medium was replenished after each withdrawal to maintain the total volume. Free curcumin, which was pre-dissolved in ethanol, was also tested as a control to determine its diffusion rate across the dialysis membrane.

### 3.11. Statistical Analysis

Statistical analysis was performed using IBM SPSS Statistics 25 (Armonk, New York, NY, USA). One-way analysis of variance (ANOVA) was used to compare samples. The significance level (*P*) was set to 0.05. All samples were performed in triplicate.

## 4. Conclusions

In this study, oxidized dextran successfully crosslinked proteins to aid stability of complex nanoparticles as evidenced by FTIR, fluorescence, and DLS data. TEM images revealed spherical nanoscale morphology with uniform size distribution for the complex nanoparticles prepared with a heating time of 60 min or longer. The hydrophobic zein protein in the core served as an ideal loading vehicle for lipophilic bioactives (curcumin was studied as a model compound) with an encapsulation efficiency as high as 95% at 10% loading ratio by weight of complex nanoparticles. The loading methods to encapsulate curcumin had a significant impact on the particulate structure of the complex nanoparticles. Pre-dissolving curcumin with zein was critical to ensure curcumin loading inside the hydrophobic core of the complex nanoparticles without a dramatic expansion of the particle size. Oxidized dextran, as a macromolecular crosslinker, significantly improved not only the colloidal stability of complex nanoparticles but also the controlled release rate of curcumin under both gastric and intestinal conditions. In addition, using oxidized dextran could potentially overcomes the toxicity concerns that arise from using synthetic and small molecular crosslinkers, and improve the redispersibility of crosslinked complex nanoparticles after spray-drying. Collectively, our results demonstrated that the use of oxidized dextran as a macromolecular crosslinker to stabilize complex protein-based nanoparticles can be a promising strategy to develop oral delivery systems for lipophilic bioactive compounds.

## Figures and Tables

**Table 1:** Particulate characteristics of nanoparticles and colloidal stability under pH 2 and pH 7.

Sample	Original		pH 2		pH 7	
	Size (nm)	PDI	Size (nm)	PDI	Size (nm)	PDI
UH1	147 ± 8	0.09 ± 0.01	571 ± 172	0.48 ± 0.10	95 ± 2	0.32 ± 0.00
UH2	152 ± 8	0.09 ± 0.01	578 ± 17	0.37 ± 0.02	94 ± 4	0.34 ± 0.05
NP1-30	150 ± 3	0.10 ± 0.02	172 ± 6	0.34 ± 0.04	100 ± 4	0.29 ± 0.01
NP2-30	148 ± 5	0.08 ± 0.02	163 ± 6	0.27 ± 0.02	104 ± 4	0.28 ± 0.01
NP1-60	143 ± 2	0.08 ± 0.01	164 ± 14	0.32 ± 0.01	103 ± 9	0.29 ± 0.01
NP2-60	150 ± 8	0.09 ± 0.02	151 ± 3	0.26 ± 0.03	105 ± 2	0.29 ± 0.01
NP1-90	149 ± 4	0.09 ± 0.02	159 ± 3	0.29 ± 0.003	108 ± 7	0.28 ± 0.01
NP2-90	146 ± 4	0.11 ± 0.01	150 ± 2	0.25 ± 0.01	110 ± 6	0.29 ± 0.00

Abbreviations: UH, zein/NaCas/oxidized dextran nanoparticles without heating; NP1−30, −60 and −90: zein/NaCas/oxidized dextran nanoparticles with heating under 80 °C for 30, 60, and 90 min, respectively.

**Table 2:** Characterizations of curcumin-loaded nanoparticles.

Loading Method	Sample	Size (nm)	PDI	Zeta Potential (mV)	Encapsulation Efficiency (%)
Method 1	Cur NP1-60	179 ± 9	0.13 ± 0.03	−46 ± 1	90 ± 0.3
	Cur-NP2-60	172 ± 6	0.13 ± 0.02	−44 ± 5	95 ± 0.8
Method 2	Cur-NP1-60	144 ± 3	0.10 ± 0.02	−43 ± 1	92 ± 0.2
	Cur-NP2-60	143 ± 1	0.11 ± 0.02	−50 ± 1	95 ± 0.5

## References

[1] Patel A.R., Velikov K.P. (2011). Colloidal delivery systems in foods: A general comparison with oral drug delivery. LWT Food Sci. Technol..

[2] Wang T., Luo Y. (2019). Biological fate of ingested lipid-based nanoparticles: Current understanding and future directions. Nanoscale.

[3] Wang T., Bae M., Lee J.Y., Luo Y. (2018). Solid lipid-polymer hybrid nanoparticles prepared with natural biomaterials: A new platform for oral delivery of lipophilic bioactives. Food Hydrocoll..

[4] Jones O.G., McClements D.J. (2011). Recent progress in biopolymer nanoparticle and microparticle formation by heat-treating electrostatic protein–polysaccharide complexes. Adv. Colloid Interf. Sci..

[5] Hosseini S.M.H., Emam-Djomeh Z., Sabatino P., Meeren P.V. (2015). Nanocomplexes arising from protein-polysaccharide electrostatic interaction as a promising carrier for nutraceutical compounds. Food Hydrocoll..

[6] Luo Y., Wang Q. (2014). Zein-based micro- and nano-particles for drug and nutrient delivery: A review. J. Appl. Polym. Sci..

[7] Bisharat L., Berardi A., Perinelli D.R., Bonacucina G., Casettari L., Cespi M., AlKhatib H.S., Palmieri G.F. (2018). Aggregation of zein in aqueous ethanol dispersions: Effect on cast film properties. Int. J. Biol. Macromol..

[8] Kasaai M.R. (2018). Zein and zein -based nano-materials for food and nutrition applications: A review. Trends Food Sci. Technol..

[9] Yuan Y., Li H., Liu C., Zhu J., Xu Y., Zhang S., Fan M., Zhang D., Zhang Y., Zhang Z. (2019). Fabrication of stable zein nanoparticles by chondroitin sulfate deposition based on antisolvent precipitation method. Int. J. Biol. Macromol..

[10] Veneranda M., Hu Q., Wang T., Luo Y., Castro K., Madariaga J.M. (2018). Formation and characterization of zein-caseinate-pectin complex nanoparticles for encapsulation of eugenol. LWT Food Sci. Technol..

[11] Luo Y., Teng Z., Wang T.T., Wang Q. (2013). Cellular uptake and transport of zein nanoparticles: Effects of sodium caseinate. J. Agric. Food Chem..

[12] Xue J., Zhang Y., Huang G., Liu J., Slavin M., Yu L.L. (2018). Zein-caseinate composite nanoparticles for bioactive delivery using curcumin as a probe compound. Food Hydrocoll..

[13] Li H., Xu Y., Sun X., Wang S., Wang J., Zhu J., Wang D., Zhao L. (2018). Stability, bioactivity, and bioaccessibility of fucoxanthin in zein-caseinate composite nanoparticles fabricated at neutral pH by antisolvent precipitation. Food Hydrocoll..

[14] Chang C., Wang T., Hu Q., Luo Y. (2017). Zein/caseinate/pectin complex nanoparticles: Formation and characterization. Int. J. Biol. Macromol..

[15] Chang C., Wang T., Hu Q., Zhou M., Xue J., Luo Y. (2017). Pectin coating improves physicochemical properties of caseinate/zein nanoparticles as oral delivery vehicles for curcumin. Food Hydrocoll..

[16] Chang C., Wang T., Hu Q., Luo Y. (2017). Caseinate-zein-polysaccharide complex nanoparticles as potential oral delivery vehicles for curcumin: Effect of polysaccharide type and chemical cross-linking. Food Hydrocoll..

[17] Hu Q., Bae M., Fleming E., Lee J.Y., Luo Y. (2019). Biocompatible polymeric nanoparticles with exceptional gastrointestinal stability as oral delivery vehicles for lipophilic bioactives. Food Hydrocoll..

[18] Wang X., Xiong Y.L. (2016). Oxidative polyaldehyde production: A novel approach to the conjugation of dextran with soy peptides for improved emulsifying properties. J. Food Sci. Technol..

[19] Oliver S., Yee E., Kavallaris M., Vittorio O., Boyer C. (2018). Water soluble antioxidant dextran–quercetin conjugate with potential anticancer properties. Macromol. Biosci..

[20] Xin Y., Yuan J. (2012). Schiff’s base as a stimuli-responsive linker in polymer chemistry. Polym. Chem..

[21] Shang J., Gao X. (2014). Nanoparticle counting: Towards accurate determination of the molar concentration. Chem. Soc. Rev..

[22] Teng Z., Luo Y., Wang Q. (2013). Carboxymethyl chitosan–soy protein complex nanoparticles for the encapsulation and controlled release of vitamin D3. Food Chem..

[23] Maia J., Ferreira L., Carvalho R., Ramos M.A., Gil M.H. (2005). Synthesis and characterization of new injectable and degradable dextran-based hydrogels. Polymer.

[24] Jamwal S., Dautoo U.K., Ranote S., Dharela R., Chauhan G.S. (2019). Enhanced catalytic activity of new acryloyl crosslinked cellulose dialdehyde-nitrilase Schiff base and its reduced form for nitrile hydrolysis. Int. J. Biol. Macromol..

[25] Acharya D.P., Sanguansri L., Augustin M.A. (2013). Binding of resveratrol with sodium caseinate in aqueous solutions. Food Chem..

[26] Wang Y.H., Wan Z.L., Yang X.Q., Wang J.M., Guo J., Lin Y. (2016). Colloidal complexation of zein hydrolysate with tannic acid: Constructing peptides-based nanoemulsions for alga oil delivery. Food Hydrocoll..

[27] Rodea-González D.A., Cruz-Olivares J., Román-Guerrero A., Rodríguez-Huezo M.E., Vernon-Carter E.J., Pérez-Alonso C. (2012). Spray-dried encapsulation of chia essential oil (*Salvia hispanica* L.) in whey protein concentrate-polysaccharide matrices. J. Food Eng..

[28] Wang T., Hu Q., Zhou M., Xia Y., Nieh M.P., Luo Y. (2016). Development of “all natural” layer-by-layer redispersible solid lipid nanoparticles by nano spray drying technology. Eur. J. Pharm. Biopharm..

[29] Vega C., Roos Y.H. (2006). Invited review: Spray-dried dairy and dairy-like emulsions—compositional considerations. J. Dairy Sci..

[30] Patel A.R., Bouwens E.C.M., Velikov K.P. (2010). Sodium caseinate stabilized zein colloidal particles. J. Agri. Food Chem..

[31] Chen H., Zhong Q. (2014). Processes improving the dispersibility of spray-dried zein nanoparticles using sodium caseinate. Food Hydrocoll..

[32] Xue J., Wang T., Hu Q., Zhou M., Luo Y. (2018). Insight into natural biopolymer-emulsified solid lipid nanoparticles for encapsulation of curcumin: Effect of loading methods. Food Hydrocoll..

[33] Wang T., Ma X., Lei Y., Luo Y. (2016). Solid lipid nanoparticles coated with cross-linked polymeric double layer for oral delivery of curcumin. Colloid Surf. B Biointerfaces.

[34] Chen Y., Xue J., Wusigale, Wang T., Hu Q., Luo Y. (2020). Carboxymethylation of phytoglycogen and its interactions with caseinate for the preparation of nanocomplex. Food Hydrocoll..

[35] Amani S., Mohamadnia Z., Mahdavi A. (2019). pH-responsive hybrid magnetic polyelectrolyte complex based on alginate/BSA as efficient nanocarrier for curcumin encapsulation and delivery. Int. J. Biol. Macromol..

[36] Xu W., Huang L., Jin W., Ge P., Shah B.R., Zhu D., Jing J. (2019). Encapsulation and release behavior of curcumin based on nanoemulsions-filled alginate hydrogel beads. Int. J. Biol. Macromol..

[37] Zhou M., Hu Q., Wang T., Xue J., Luo Y. (2018). Alginate hydrogel beads as a carrier of low density lipoprotein/pectin nanogels for potential oral delivery applications. Int. J. Biol. Macromol..

[38] Muangsiri W., Kirsch L.E. (2006). The protein-binding and drug release properties of macromolecular conjugates containing daptomycin and dextran. Int. J. Pharm..

[39] Pan K., Luo Y., Gan Y., Baek S.J., Zhong Q. (2014). pH-driven encapsulation of curcumin in self-assembled casein nanoparticles for enhanced dispersibility and bioactivity. Soft Matter.

[40] Wang T., Xue J., Hu Q., Zhou M., Luo Y. (2017). Preparation of lipid nanoparticles with high loading capacity and exceptional gastrointestinal stability for potential oral delivery applications. J. Colloid Interface Sci..

[41] Kakkar V., Singh S., Singla D., Kaur I.P. (2011). Exploring solid lipid nanoparticles to enhance the oral bioavailability of curcumin. Mol. Nutr. Food Res..

